# Monitoring and Modeling the Tibetan Plateau’s climate system and its impact on East Asia

**DOI:** 10.1038/srep44574

**Published:** 2017-03-13

**Authors:** Yaoming Ma, Weiqiang Ma, Lei Zhong, Zeyong Hu, Maoshan Li, Zhikun Zhu, Cunbo Han, Binbin Wang, Xin Liu

**Affiliations:** 1 Key Laboratory of Tibetan Environment Changes and Land Surface Processes, Institute of Tibetan Plateau Research, Chinese Academy of Sciences, Beijing 100101, China; 2 CAS Center for Excellence in Tibetan Plateau Earth Sciences, Chinese Academy of Sciences, Beijing 100101, China; 3 University of Chinese Academy of Sciences, Beijing 100049, China; 4 Laboratory for Atmospheric Observation and Climate Environment Research, School of Earth and Space Sciences, University of Science and Technology of China, Hefei, China; 5 Northwest Institute of Eco-Environment and Resources, Chinese Academy of Sciences, Lanzhou 730000, China; 6 Chengdu University of Information Technology, Chengdu 610225, China

## Abstract

The Tibetan Plateau is an important water source in Asia. As the “Third Pole” of the Earth, the Tibetan Plateau has significant dynamic and thermal effects on East Asian climate patterns, the Asian monsoon process and atmospheric circulation in the Northern Hemisphere. However, little systematic knowledge is available regarding the changing climate system of the Tibetan Plateau and the mechanisms underlying its impact on East Asia. This study was based on “water-cryosphere-atmosphere-biology” multi-sphere interactions, primarily considering global climate change in relation to the Tibetan Plateau -East Asia climate system and its mechanisms. This study also analyzed the Tibetan Plateau to clarify global climate change by considering multi-sphere energy and water processes. Additionally, the impacts of climate change in East Asia and the associated impact mechanisms were revealed, and changes in water cycle processes and water conversion mechanisms were studied. The changes in surface thermal anomalies, vegetation, local circulation and the atmospheric heat source on the Tibetan Plateau were studied, specifically, their effects on the East Asian monsoon and energy balance mechanisms. Additionally, the relationships between heating mechanisms and monsoon changes were explored.

Known as the Asian water tower, the Tibetan Plateau is an important water source in Asia. As the “Third Pole” of the Earth, the Tibetan Plateau has significant dynamic and thermal effects on East Asian climate patterns, the Asian monsoon process and atmospheric circulation in the Northern Hemisphere. The Tibetan Plateau has undergone extreme changes in the context of global climate change. However, little systematic knowledge is available regarding the changing climate system of the Tibetan Plateau and the mechanisms underlying its impact on East Asia. Specifically, what are the characteristics of the climate system variations on the plateau in the context of global climate change? How do changes in the Tibetan Plateau’s climate system affect East Asia? What are the underlying mechanisms? Given these questions, a Chinese national key research program, “The Study of the Changes in the Tibetan Plateau’s Climate System and its Impact on East Asia (TPCSIEA)”, was launched in 2010. A unique, integrated network of “water-cryosphere-atmosphere-biology” interactions was established within the framework of TPCSIEA. The powerful dynamics and thermal effects of the Tibetan Plateau significantly affect the East Asian climate pattern, the Asian monsoon process and atmospheric circulation in the Northern Hemisphere. Tibetan Plateau studies can further our understanding of climate change, especially on the Tibetan Plateau and in the surrounding areas. The Tibetan Plateau is one of the world’s most sensitive areas; consequently, high-quality climate change research in the area, including *in situ* observations, can provide important contributions to studies of global climate change.

## Key scientific objectives

This study was based on “water-cryosphere-atmosphere-biology” multi-sphere interactions. We focus on global climate change in relation to the Tibetan Plateau -East Asia climate system and its mechanisms. This study analyzed the Tibetan Plateau to clarify global climate change by considering multi-sphere energy and water processes to reveal the impacts of climate change on East Asia and the associated mechanisms. Changes in the water cycle and water conversion mechanisms were studied. The Tibetan Plateau’s surface thermal anomalies, vegetation changes, local circulation and atmospheric heat source changes that affect the East Asian monsoon and energy balance mechanisms were also studied. Additionally, the relationships between heating mechanisms and monsoon changes were explored. The following three key scientific issues were solved:

1. Improved understanding of global climate change, hydrosphere, cryosphere and ecosystem responses and their impacts on atmospheric circulation anomalies on the Tibetan Plateau. The main scientific problem was based on the influence and response of atmospheric circulation anomalies according to the multi-sphere energy exchange processes. To obtain detailed information regarding land surface characteristics, an integrated observation and data collection network for multi-sphere interactions was set up across the Tibetan Plateau and the surrounding areas.
2. To document the observed changes of climate on the Tibetan Plateau. According to *in situ*, reanalysis and remote sensing data, a synthesis of results about land surface temperature (LST), surface air temperature, soil moisture, vegetation cover and radiative and turbulent heat fluxes are presented in this paper.
3. The mechanism of the change in the Tibetan Plateau climate system influences the East Asia climate system. The following major scientific problems were analyzed: the relationship between climate system variations in East Asia and global climate change; the manners in which the East Asian monsoon is influenced by land, surface, water and energy anomalies, vegetation changes, atmospheric heating source changes and the local atmospheric cycle; and the rapid warming mechanism of the surface energy balance and its interactions with the Tibetan Plateau -Asia monsoon. The Tibetan Plateau also has an impact on East Asian monsoons. Observations and climate proxies, satellite remote sensing data, data assimilation techniques and climate models were used to study the Tibetan Plateau, consider the relationship between change in the East Asian climate system and global change and analyze recent key factors in Tibetan Plateau spheres (atmospheric heat, soil moisture, snow cover and vegetation) and basic abnormal changes in East Asian monsoon characteristics. The East Asian region has certain advantages as a simulated climate system model; consequently, we were able to simulate the key factors and mechanisms that influence East Asian monsoon process anomalies and cause the rapid heating of the Tibetan Plateau energy balance mechanism. Additionally, we explore the interaction between the Tibetan Plateau and the warming of the Asian monsoon.

## The construction of a unique integrated observation network for “water-cryosphere-atmosphere-biology” multi-sphere interactions

**Figure 1:**
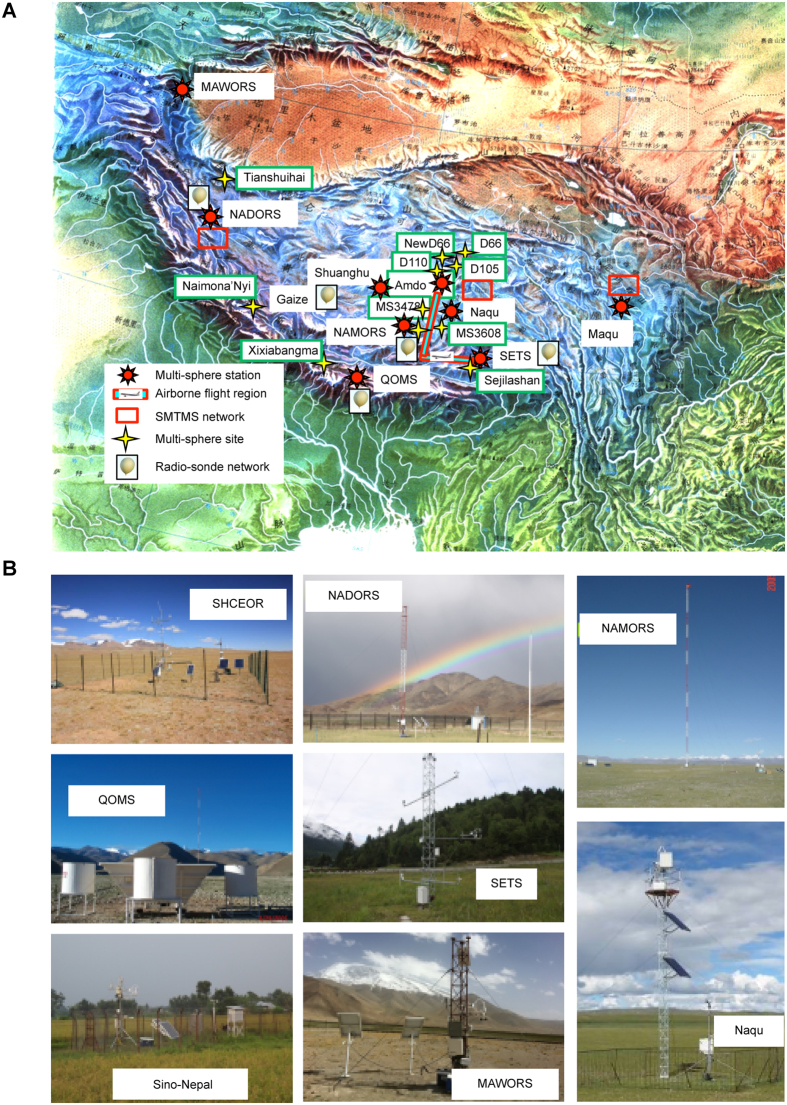
Spatial distribution of observation stations (sites) of water-cryosphere-atmosphere-biology interactions over the Tibetan Plateau (**A**) and the typical observation instruments for the different land surfaces (**B**). The Fig. 1(A) was produced using ArcGIS 9.3 (http://www.esri.com/arcgis/about-arcgis) and NCL (The NCAR Command Language (Version 6.3.0) [Software]. (2016). Boulder, Colorado: UCAR/NCAR/CISL/TDD. http://dx.doi.org/10.5065/D6WD3XH5). All the figures were created by Dr. Weiqiang Ma and Dr. Lei Zhong.

A unique integrated network platform for “water-cryosphere-atmosphere-biology” multi-sphere interactions was created in the TPCSIEA framework (Fig. 1). It includes 10 multi-sphere stations, 21 air-land flux exchange stations, 10 multi-sphere sites, 3 soil moisture and soil temperature networks, 5 radiosonde stations, 6 glacier monitoring sites, 3 lake monitoring sites and 8 isotope monitoring sites. All instruments performed continuous observations for more than 5-and-a-half years. The monitoring network covers most parts of the Tibetan Plateau, and the observation period is long and continuous, including an intensive observation period from May to October 2011. More than 100 sets of instruments and 120 staff members (scientists, engineers and students) were involved in the field experiments. Typical land surface types are plateau mountain, desertification grass-land, plateau forest, plateau gobi desert, grass land, glacier (snow mountain), plateau lake, farm-land, wet-land, for more in detail in Fig. 1B. Large amounts of hydrometeorology, soil, vegetation, snow, glacier, social and economic data were collected to create a database for the study of land-atmosphere interactions and energy and water cycling over the Tibetan Plateau, as well as their weather, climate, socioeconomic, ecological and environmental impacts in East Asia.

## Monitoring the changing climate over the Tibetan Plateau

Some scientific breakthroughs have been achieved through combinations of *in situ* measurements and satellite observations. Global surface temperatures increased by approximately 0.85 °C (0.65 to 1.06 °C) between 1880 and 2012, and the linear increase in temperature over the past 50 years was 0.12 °C per decade (0.08 to 0.14 °C), which is nearly twice that of the past 100 years^1^,^2^. However, warming has not been globally uniform. Both optical and passive microwave remote sensing data were used to study land surface temperature variations over the Tibetan Plateau^3^,^4^,^5^. Recent analyses using *in situ*, reanalysis and satellite data have demonstrated that the Tibetan Plateau has warmed more than other parts of China^4^,^6^. The results indicate that both the LST and the surface air temperature increased on the Tibetan Plateau from 1982 to 2000. The rate of the increase in the LST was 0.26 ± 0.16 K decade^−1^, and that of the surface air temperature was 0.29 ± 0.16 K decade^−1^. Both exceeded the increase in the Northern Hemisphere (0.054 K decade^−1^). The highest positive trends were noted over the central part of the Plateau while negative anomalies can also be found at Taklamakan desert and the Himalayan foothills^5^. Accordingly, the surface temperature rise will cause the retreat of permafrost and it could be one of the factors contributing to the wetting over the Tibetan Plateau. The wetting trend of the Tibetan Plateau has been proved from Microwave perspective^7^,^8^. It was found that the central Tibetan Plateau has the most severe wetting trend. Under the background of Tibetan Plateau warming and wetting, the vegetation density was firstly found increasing in 49.87% of the total Tibetan Plateau area^9^. A spatially and temporally explicit estimate of surface energy fluxes is of considerable interest for hydrological meteorological and climatological investigations. Based on Surface Energy Balance Model (SEBS)^10^, a 10-year dataset of land surface energy balance was developed^11^. Trend analysis of land-surface radiation and energy exchange fluxes revealed that the Tibetan Plateau has undergone relatively stronger climatic change than other parts of China during 2001 and 2010.

**Figure 2:**
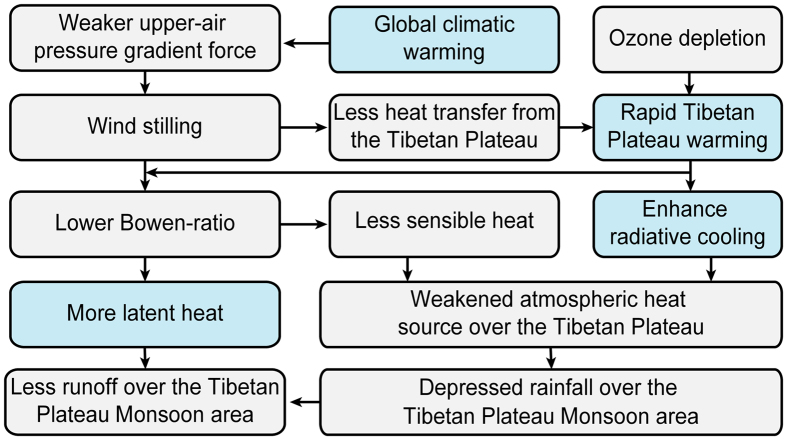
A conceptual model showing changing climate elements over the Tibetan Plateau in response to global climate warming.

Due to asymmetrical warming between lower and higher latitudes, a weaker upper-air pressure gradient force (PGF) occurred. Both modeling results^12^ and observational evidence^13^ suggest that the weaker PGF will eventually decrease the surface wind speed over the Tibetan Plateau through a downward movement into the atmospheric boundary layer. The warming rate over the Tibetan Plateau will increase faster than in other places because wind stilling will decrease the amount of heat transferred from the Tibetan Plateau, as well as cause ozone depletion, CO_2_ effects and the enrichment of surface water vapor (Fig. 2). Furthermore, with the rapid retreat and thinning of permafrost, large carbon pools sequestered in permafrost could be released to increase net sources of atmospheric carbon, creating a positive feedback cycle and accelerated warming^14^.

The rapid warming and wind stilling will lower the Bowen ratio and reduce the surface sensible heat flux. Moreover, warming will enhance atmospheric radiative cooling by outgoing longwave radiation. Both processes contributed to the thermal forcing weakening (−1.2 W m^−2^ decade^−1^)^15^ over the Tibetan Plateau, which in turn may have contributed to weakening the Asia monsoon.

Research has found that the water cycle and its related elements are altered by Tibetan Plateau climate changes^6^. The southern and eastern Tibetan Plateau are experiencing water shortages, whereas the central Tibetan Plateau has more water resources. Wind stilling over the Tibetan Plateau may have weakened water vapor exchange between the Asian monsoon region and the Tibetan Plateau, leading to less precipitation in the monsoon-impacted southern and eastern parts of the Tibetan Plateau. Additionally, warming has increased land evaporation. These trends have decreased runoff in the southern and eastern Tibetan Plateau regions. By contrast, more convective precipitation has occurred over the central Tibetan Plateau under warmer and moister conditions, which have yielded more runoff. Meanwhile, solar dimming has decreased lake evaporation. These two factors, in addition to enhanced glacier melting, have contributed to lake expansion on the central Tibetan Plateau. All these changes of environmental conditions caused by climate change will also have thorough impacts on the shifting patterns of different sub-regions of the Tibetan Plateau especially for the arid and semi-arid area^16^,^17^.

## Modeling the Tibetan Plateau’s feedback in East Asia

**Figure 3:**
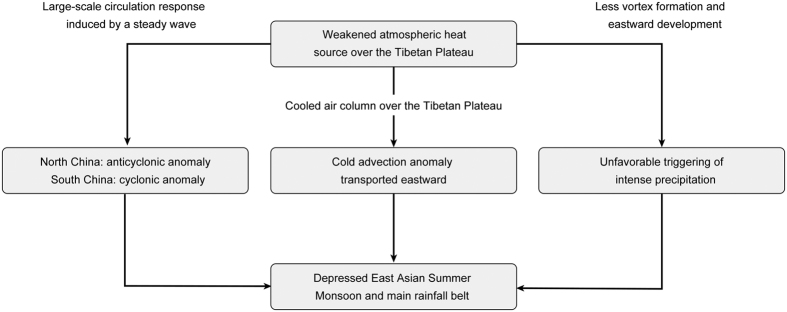
The inner mechanisms between the atmospheric heat source and its impacts on the EASM.

For the time being, the role of the Tibetan Plateau in the Asian monsoon system remains a topic of debate^18^,^19^. One fundamental reason for the lack of agreement is the lack of climate observations on the Tibetan Plateau, especially regarding the land-atmosphere flux exchange, which inhibits the predictive power of global climate models of the region^20^. Therefore, based on satellite and *in situ* network data, the aerodynamic and thermodynamic parameters (such as the aerodynamic roughness length, thermodynamic roughness length and zero-plane displacement) over different underlying surfaces were determined. A parameterization scheme was proposed and tested to determine the regional surface heating field, sensible heat flux and latent heat flux over the plateau-scale heterogeneous landscape^21^. Both *in situ* measurements and satellite results indicated that the surface sensible heat flux on the Tibetan Plateau is decreasing^15^,^22^. Further analysis revealed that the reductions in the sensible heat flux over the Tibetan Plateau have weakened monsoon circulation and postponed the seasonal reversal of the land-sea thermal contrast in East Asia^23^. The time-lagged impacts of the spring sensible heat source over the Tibetan Plateau in East China were investigated using the Weather Research and Forecasting model^24^. The WRF model was used in this study. Physical packages include the cloud microphysics scheme, the convective scheme, the NOAH land-surface model, the planetary boundary layer (PBL) scheme, a shortwave scheme, and the Rapid Radiative Transfer Model (RRTM) for longwave radiation. The simulation domain covers most parts of Asia and adjacent oceans. The initial state of the atmosphere and lateral boundary conditions are from NCEP-FNL^24^. The spring sensible heat anomaly over the Tibetan Plateau was found to have a considerable influence on the inter-annual variability of the East Asian Summer Monsoon (EASM) in terms of wave activity and synoptic disturbance. Additionally, following inner mechanisms were revealed (Fig. 3). First, a wave activity analysis demonstrated that the weakened atmospheric heating source over the Tibetan Plateau could cause large-scale atmospheric circulation responses induced by steady waves. There are anticyclonic anomalies in North China and cyclonic anomalies in South China. Therefore, no favorable atmospheric circulations will occur around the Huaihe River Basin. Second, the weakened heat source over the Tibetan Plateau will cool the local air column. Thus, the cold advection anomaly will be transported eastward. Third, the weakened heat source over the Tibetan Plateau will lead to less vortex formation and eastward development, which will be unfavorable for triggering intense precipitation. All these factors will eventually suppress the EASM and main rainfall belt.

## Summary

This study focused on “water-cryosphere-atmosphere-biology” interactions by treating the Tibetan Plateau multi-sphere as a climate system and investigated the mechanisms of its development and the effects on East Asia in the context of global change. Both the impacts of global change and feedback from the Tibetan Plateau were combined in this study. Based on comprehensive, continuous, intensive observations of “water-cryosphere-atmosphere-biology” interactions (combining spaceborne and airborne remote sensing technology), a multi-sphere interaction database has been established for the whole Tibetan Plateau and some key regions. The establishment of the database benefits the study of the climate system change of the Tibetan Plateau and its impacts on the East Asian region. The changing characteristics and mechanisms of the Tibetan Plateau climate system, such as rapid warming, wind stilling and water cycle changes, were systematically revealed. The aerodynamic and thermodynamic parameters were determined over different surface layer types. Then, a parameterization scheme based on satellite data was proposed and tested to derive the regional surface heating field, sensible heat flux and latent heat flux over the plateau-scale heterogeneous landscape. Based on these findings, the early stage thermal conditions on the Tibetan Plateau and their linkage with East Asian climate system were the focus of this study. We found that the weakening of the East Asian monsoon in recent decades was mainly caused by the reduction in the spring sensible heat flux on the Tibetan Plateau. Both *in situ* observations and model simulations indicated that the abnormal signal of spring sensible heat flux over the Tibetan Plateau can lead to a general anomaly of the atmospheric heating source through the positive feedback of atmospheric circulation-diabatic heating. It will further affect the downstream circulation and precipitation in East Asia through large-scale steady waves, warm advection and vortex disturbances. The findings provide a theoretical basis and prediction method for the study of East Asian summer climate variability.

## Additional Information

**How to cite this article:** Ma, Y. *et al*. Monitoring and Modeling the Tibetan Plateau’s climate system and its impact on East Asia. *Sci. Rep.* **7**, 44574; doi: 10.1038/srep44574 (2017).

**Publisher's note:** Springer Nature remains neutral with regard to jurisdictional claims in published maps and institutional affiliations.
